# Broadening the Mutation Spectrum in *GJA8* and *CHMP4B*: Novel Missense Variants and the Associated Phenotypes in Six Chinese Han Congenital Cataracts Families

**DOI:** 10.3389/fmed.2021.713284

**Published:** 2021-10-15

**Authors:** Xun Wang, Dongni Wang, Qiwei Wang, Weiming Huang, Meimei Dongye, Xulin Zhang, Duoru Lin, Zhuoling Lin, Jing Li, Weiling Hu, Xiaoyan Li, Xiaoshan Lin, Qiuping Zhong, Weirong Chen, Haotian Lin

**Affiliations:** ^1^State Key Laboratory of Ophthalmology, Zhongshan Ophthalmic Center, Sun Yat-sen University, Guangzhou, China; ^2^Center for Precision Medicine, Sun Yat-sen University, Guangzhou, China

**Keywords:** *CRYBB2*, *GJA8*, *CHMP4B*, congenital cataracts, whole-exome sequencing, phenotype-genotype correlation

## Abstract

**Purpose:** To broaden the mutation and phenotype spectrum of the *GJA8* and *CHMP4B* genes and to reveal genotype-phenotype correlations in a cohort of Chinese patients with congenital cataracts (CCs).

**Methods:** Six Chinese Han families with CCs inherited in an autosomal dominant (AD) pattern were recruited for this study. All patients underwent full ocular examinations. Genomic DNA was extracted from the leukocytes of peripheral blood collected from all available patients and their unaffected family members. Whole-exome sequencing (WES) was performed on all probands and at least one of their parents. Candidate variants were further confirmed by Sanger sequencing. Bioinformatic analysis with several computational predictive programs was performed to assess the impacts of the candidate variants on the structure and function of the proteins.

**Results:** Four heterozygous candidate variants in three different genes (*CRYBB2, GJA8*, and *CHMP4B*) were identified in affected individuals from the six families, including two novel missense variants (*GJA8*: c.64G > C/p. G22R, and *CHMP4B*: c.587C > G/p. S196C), one missense mutation (*CRYBB2*: c.562C > T/p. R188C), and one small deletion (*GJA8*: c.426_440delGCTGGAGGGGACCCT/p.143_147delLEGTL). The three missense mutations were predicted as deleterious in all four computational prediction programs. In the homologous model, the *GJA8*: p.143_147delLEGTL mutation showed a sequence deletion of five amino acids at the cytoplasmic loop of the Cx50 protein, close to the third transmembrane domain. Patients carrying mutations in the same gene showed similar cataract phenotypes at a young age, including total cataracts, Y-sutural with fetal nuclear cataracts, and subcapsular cataracts.

**Conclusion:** This study further expands the mutation spectrum and genotype-phenotype correlation of *CRYBB2, GJA8*, and *CHMP4B* underlying CCs. This study sheds light on the importance of comparing congenital cataract phenotypes in patients at the same age stage. It offers clues for the pathogenesis of CCs and allows for an early prenatal diagnosis for families carrying these genetic variants.

## Introduction

Congenital cataracts (CCs) are defined as an opacity of the lens with onset from birth. It is one of the most common avoidable causes of visual impairment and blindness in children worldwide (1). It accounts for ~14% of all cases of global blindness in childhood (2). According to statistical analysis, hereditary cataracts account for 22.3% of global childhood cataracts (3). To date, more than 100 genes and 200 loci associated with CCs have been identified (http://cat-map.wustl.edu/) (4). With this many causative genes and loci, it has been widely accepted that CCs are highly phenotypically and genotypically heterogeneous. Patients who carry the same mutations might have various types of lens morphologies, and different mutations can lead to similar phenotypes (5). In our previous study, a modified CC category system was proposed based on the relationships among the locations of lens opacities and anterior segment characteristics (6). Further exploration of the genotype-phenotype correlation of CC patients can help guide the screening of candidate variants and provide prenatal diagnosis of families with CC patients. In this study, four mutations of the crystallin βB2 gene (*CRYBB2*), the gap junction protein alpha 8 gene (*GJA8*), and the charged multivesicular body protein 4B gene (*CHMP4B*), were identified in six Chinese Han families with CCs. Similar cataract patterns were observed in patients carrying the same mutations or different mutations in the same genes. Although CCs are a phenotypically heterogeneous disease, genotype-phenotype correlation analysis of cataract morphology may allow for more efficient genetic and prenatal diagnosis of families carrying these genetic alterations. It could also reveal the cataract genesis mechanisms of candidate genes in the different stages of lens development.

## Materials and Methods

### Subjects

**Figure 1:**
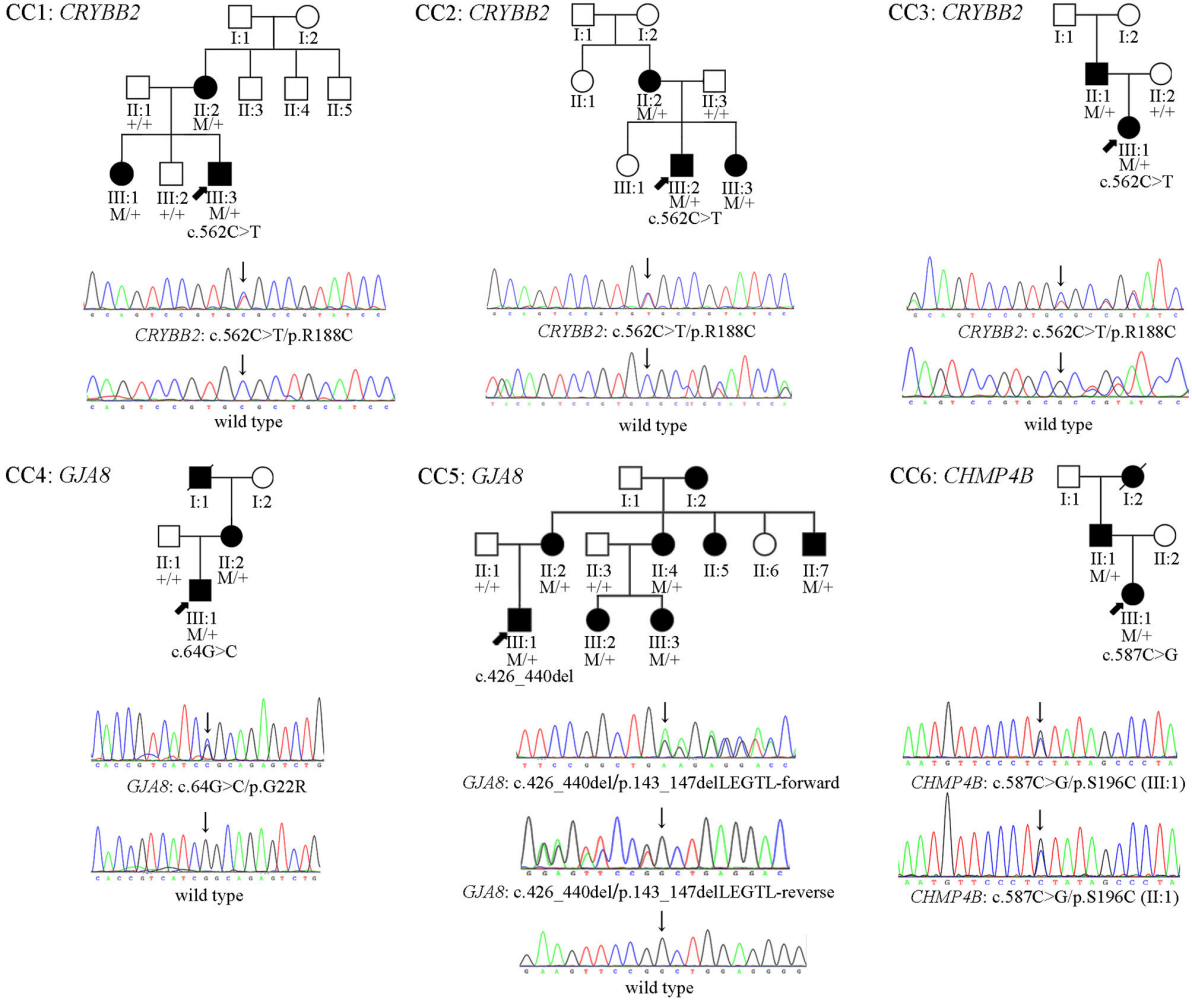
The pedigrees, and cosegregation analyses and sequence maps of six Chinese Han families of CCs. In family CC1–CC5, the sequence maps are for the probands and their unaffected parents. In family CC6, the sequence map is for the proband and her affected father.

A total of 25 participants (18 patients and seven unaffected relatives) in six Chinese families (Figure 1) were recruited from the Home for Cataract Children of Zhongshan Ophthalmic Center between January 2020 and November 2020. This study was approved by the Institutional Review Board of Zhongshan Ophthalmic Center (2020KYPJ007) and was carried out following the rules of the Declaration of Helsinki of 1975 (https://www.wma.net/what-we-do/medical-ethics/declaration-of-helsinki/), revised in 2013. Informed consent was provided by all of the participants or their guardians. Detailed histories of birth conditions and ophthalmology were collected. A full ophthalmic examination was performed on all available subjects. It included a best-corrected visual acuity assessment, slit-lamp biomicroscopy, fundus photography, B-ultrasound scan, retinoscopy with cycloplegia, anterior segment biometry measurements performed with a Pentacam rotating Scheimpflug camera (Oculus, Wetzlar, Germany), and ocular biometry measurements performed with an A-ultrasound scan or an IOL Master 500 (Carl Zeiss Meditec AG, Jena, Germany).

### Mutation Screening

Genomic DNA was extracted from leukocytes obtained from peripheral blood samples, as previously described (7). Whole-exome sequencing (WES) technology was carried out on all probands from the six families and their available family members. The Agilent SureSelect Human All Exon V6 Kit (58 Mb) was used for targeted enrichment of exomes (Agilent Technologies Inc., CA, USA). The mean read depth of all samples ranged from ~50 X, and the average read quality was well above the standard of 20 X. We conducted paired-end 2 × 150 bp sequencing on an Illumina NovaSeq 6000 platform (Illumina Inc., CA, USA). Raw sequencing reads were mapped to the human reference genome assembly (NCBI build 37/hg19) using Burrows-Wheeler Aligner (8). The Genome Analysis Toolkit (GATK) was used for local realignment, base quality score recalibration and variant calling (HaplotypeCaller) (9). To effectively remove sites with low quality, we filtered out sample genotypes with a coverage <8 × , a genotype quality <20 and a call rate ≤ 0.90. The remaining variants were annotated by the ANNOVAR tool (10). Sanger sequencing was used to validate the variants detected in WES. See Supplementary Table 1 for all primers used for amplification and sequencing. Segregation analyses of the mutations were performed for all available family members.

### Pathogenicity Assessment of CC Variants

The variants detected through WES were filtered by multistep bioinformatics analyses. The frequencies of the variants were determined from the 1,000 Genomes database (http://grch37.ensembl.org/Homo_sapiens/Info/Index, Sep. 2015 data release), the dbSNP database (https://www.ncbi.nlm.nih.gov/snp/, Jun. 2020 data release), and the gnomAD database (http://gnomad.broadinstitute.org/, Oct. 2018 data release). All alleles with minor allele frequencies (MAFs) larger than 0.0002 were removed. The frequency was based on the incidence of CCs (2.2/10,000) (3). The pathogenicity of variants in the exons was evaluated by bioinformatics prediction tools, including PolyPhen-2 (http://genetics.bwh.harvard.edu/pph2/index.shtml) (11), SIFT and PROVEAN (http://provean.jcvi.org/index.php) (12), and MutationTaster (http://www.mutationtaster.org/) (13). The pathogenicity of the variants in the introns and synonymous variants in the exons were predicted by the Splice Site Prediction program with Neural Network (BDGP, https://www.fruitfly.org/seq_tools/splice.html) (14). The evaluation of cross-species conservation was performed by multiple protein sequence alignments in the UCSC Genome Browser (https://genome.ucsc.edu/index.html). The filtered results were analyzed in the Human Gene Mutation Database (HGMD, http://www.hgmd.cf.ac.uk/ac/index.php, last updated on Mar. 2020) and the Cat-Map database (http://cat-map.wustl.edu, last updated on Nov. 2020) to confirm whether the candidate variants we detected had been reported before. All candidate variants were further filtered by the phenotypes of the patients carrying these mutations and the cosegregation analysis. The ProtScale program (https://web.expasy.org/protscale/) was used to predict protein hydrophobicity. In addition, homologous structures of wild-type and mutant human βB2-crystalline protein, connexin 50 (Cx50), and chromatin-modifying protein 4b were modeled by the SWISS-MODEL program based on the template of the resolved structure of No. 1blb, 7jjp, and AF-Q9H444-F1 (https://beta.swissmodel.expasy.org/) (15).

## Results

### Molecular Findings

Four heterozygous mutations spread in three different genes (*CRYBB2, GJA8*, and *CHMP4B*) were identified in affected individuals from six Chinese Han families. Two variants were novel: *GJA8* (OMIM: 600897): c.64G > C (p. G22R), and *CHMP4B* (OMIM: 610897): c.587C > G (p.S196C). The other mutants included a missense mutation: *CRYBB2* (OMIM: 123620): c.562C > T (p. R188C), and a small deletion: *GJA8* (OMIM: 600897): c.426_440delGCTGGAGGGGACCCT (p.143_147delLEGTL), which has been previously reported in other Chinese families (16, 17). All candidate variants were predicted to damage the function of the encoded protein by the online tools PolyPhen-2 (11), SIFT, PROVEAN (12), and MutationTaster (13) (Table 1). All mutations were absent in the 1,000 Genomes database, the dbSNP database, and the gnomAD database (Table 1). The pedigrees, cosegregation analyses, and sequence maps of the six families are shown in Figure 1. The detailed information, MAF, and computational prediction results of the four candidate variants are summarized in Table 1. Other variants that were detected in the known eye disease-causing genes in the six probands and the reasons for excluding them are listed in Supplementary Table 2.

**Table 1:** Mutations in the *CRYBB2, GJA8*, and *CHMP4B* genes were identified in six Chinese Han families with CCs.

**Family ID**	**Gene**	**Position (GRCh37/hg19)**	**Exon**	**Base change**	**Amino acid change**	**Inheritance patterns**	**MAF (%) in 1000G/dbSNP/gnomAD**	**PolyPhen-2/SIFT/PROVEAN/Mutation Taster**	**Reported previously**
CC1/CC2/CC3	*CRYBB2*	chr22:25627683	6	c.562C>T	p.R188C	AD	NA/NA/NA	PrD/D/D/DC	(16)
CC4	*GJA8*	chr1:147380146	2	c.64G>C	p.G22R	AD	NA/NA/NA	PrD/D/D/DC	No
CC5	*GJA8*	chr1:147380508-147380522	2	c.426_440delGCTGGAGGGGACCCT	p.143_147delLEGTL	AD	NA/NA/NA	-	(17)
CC6	*CHMP4B*	chr20:32439986	4	c.587C>G	p.S196C	AD	NA/NA/NA	PrD/D/D/DC	No

*Chr, chromosome; AD, autosomal dominant; NA, these variants were absent from the 1,000 Genome, the dbSNP, and the gnomAD databases; PrD, probably damaging; D, damaging in SIFT and deleterious in PROVEAN; DC, Disease causing; -, not applicable*.

**Figure 2:**
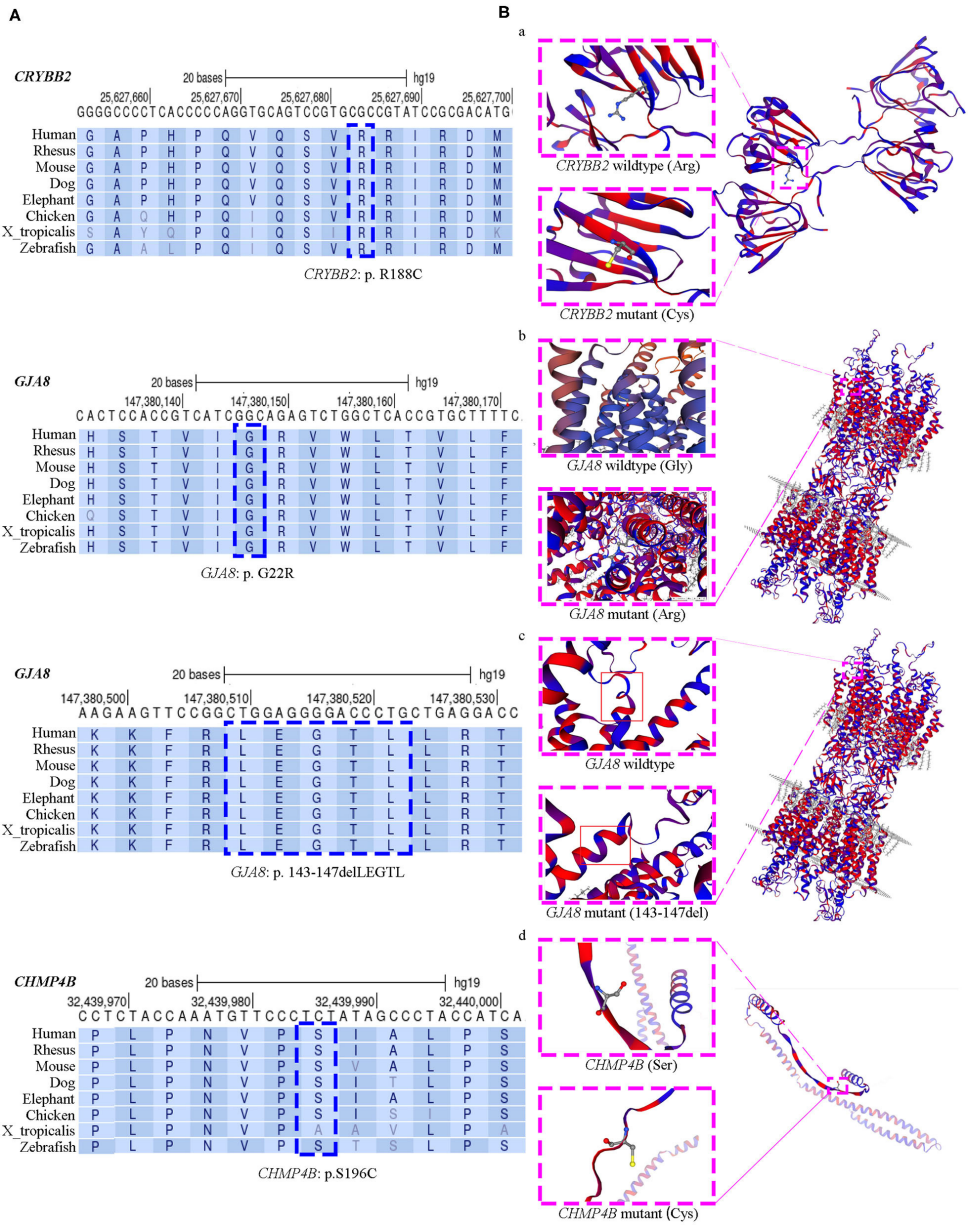
Evolutionary sequence conservation analysis and structure homology modeling of mutant proteins and native proteins. **(A)** Multiplex sequence alignment of the βB2-crystalline protein, connexin 50, and the chromatin-modifying protein 4b from different species reveals that the four mutation (*CRYBB2*: R188C, *GJA8*: G22R, *GJA8*: p.143_147del LEGTL, and *CHMP4B*: S196C) are located within highly conserved regions (blue dashed box). **(B)** Structure homology modeling and comparison of mutant protein and native βB2-crystalline protein, Cx50, and the chromatin-modifying protein 4b (pink dashed box). The secondary structure was shown with α-helixes, β-sheets and loops in hydrophobic color scheme. From purple to red shows the least hydrophobic residues to most hydrophobic residues. (a) In the missense *CRYBB2*: p.R188C, the substitution replaces a basic arginine residue with an uncharged cysteine residue at the surface of the βB2-crystalline protein; (b) In the missense *GJA8*: p.G22R, a buried uncharged glycine residue mutants to a charged arginine residue, which located at the first transmembrane region of Cx50; (c) The small deletion *GJA8*: p.143_147delLEGTL locates at the cytoplasmic loop of the Cx50 protein, close to the third transmembrane domain; (d) The *CHMP4B*: p.S196C mutation is a substitution of serine residue to cysteine residue, which is located in a random coil region. This mutation makes a new exposed sulfhydryl group.

All four affected genetic sites are highly conserved across eight different species, including mammals, chickens, frogs, and zebrafish (Figure 2A). The missense mutation *CRYBB2*: p. R188C occurred de novo in families CC1, CC2, and CC3. In this mutation, the substitution replaces a basic arginine residue with an uncharged cysteine residue at the surface of the βB2-crystalline protein (Figure 2B, a). The prediction results of the ProtScale program showed that this variant has substantially higher hydrophobicity than the wild-type gene (Supplementary Figure 1). The variant *GJA8*: p. G22R is a missense mutation of a buried uncharged hydrophobic residue (glycine) to a positively charged hydrophilic residue (arginine). It is located in the first transmembrane region of the Cx50 protein (Figure 2B, b). For the mutation *GJA8*: p.143_147delLEGTL, the homologous model showed a sequence deletion of five amino acids in the cytoplasmic loop (CL) of the Cx50 protein, close to the third transmembrane domain (Figure 2B, c). The *CHMP4B*: p. S196C mutation is a substitution of a serine residue to a cysteine residue, which leads to a newly exposed sulfhydryl group. The homologous model of chromatin-modifying protein 4b showed that residue 196 is located in a random coil region (Figure 2B, d), but the model confidence of this residue is rated as very low (confidence score: 44.5).

### Clinical Data

We investigated 18 patients (11 females and seven males) with CCs and seven unaffected family members (1 female and six males) from six Chinese Han families. All patients had bilateral cataracts and a family history of autosomal dominant (AD) inheritance patterns. None of them reported cataract-causing situations during pregnancy, including virus infection (toxoplasmosis, rubella, cytomegalovirus, and herpes simplex), histories of medication use, toxin, or X-ray exposure. None of the unaffected family members carried the candidate variants. A summary of the general information and ocular phenotypes of all available patients in the six families is shown in Table 2.

**Table 2:** Clinical characteristics of affected members in the six Chinese Han families.

**Family ID**	**Inheritance patterns**	**Mutation**	**Individual ID**	**Gender**	**Age at**			**Lens**	**Axis length at last-time examination (OD/OS mm)**	**Other ocular abnormities**
					**Last examination**	**Cataract presentation**	**Cataract surgery**			
CC1	AD	CRYBB2: p.R188C	II:2	F	40 y	15 y	17 y	Pseudophakia OU	30.22/30.93	High myopia related fundus change OU
			III:1	F	3 y	5 m	5 m	Total cataracts OU	22.75/22.64	-
			III:3 (proband)	M	8 y	2 m	2 m	Total cataracts OU	23.68/22.97	Sensory esotropia OD; Nystagmus OU
CC2	AD	CRYBB2: p.R188C	II:2	F	31 y	10 m	10 m	Aphakia OU	26.73/23.98	Anisometropia OU; Sensory esotropia OD; High myopia related fundus change OD; Nystagmus OU
			III:2 (proband)	M	5 y	4 m	4 m	Total cataracts OU	20.47/19.82	-
			III:3	F	3 y 5 m	3 m	4 m	Total cataracts OU	18.5/18.42	Concomitant exotropia OS
CC3	AD	CRYBB2: p.R188C	II:1	M	33 y	<1 y	<1 y	Aphakia OU	27.28/NA	Retinal detachment OS
			III:1 (proband)	F	3 y 3 m	3 m	3 m	Total cataracts OU	22.61/23.09	Primary iris cysts OU
CC4	AD	GJA8: p.G22R	II:2	F	27 y	<1 y	<1 y	Pseudophakia OU	NA/NA	-
			III:1 (proband)	M	4 m	4 m	4 m	Y-sutural with fetal nuclear cataracts OU	22.90/23.30	-
CC5	AD	GJA8: p.143_147delLEGTL	II:2	F	32 y	<1 y	23 y	Pseudophakia OU	27.84/27.34	High myopia related fundus change OU
			II:4	F	29 y	<1 y	15 y	Pseudophakia OU	27.21/25.07	High myopia related fundus change OU
			II:7	M	23 y	6 m	3 y	Pseudophakia OU	22.77/22.98	Sensory esotropia OS; nystagmus OU
			III:1 (proband)	M	6 y	2 y 3 m	2 y 3 m	Y-sutural (dense shape) with embryonal nuclear opacities OU	23.25/22.99	Nystagmus OU
			III:2	F	10 y	2 y 9 m	5 y 10 m	Y-sutural (faint shape) with embryonal nuclear opacities OU	21.21/21.34	-
			III:3	F	5 y 3 m	10 m	10 m	Y-sutural (feathery shape) with embryonal nuclear opacities OU	25.22/25.98	High myopia related fundus change OU; Nystagmus OU
CC6	AD	CHMP4B: p.S196C	II:1	M	33 y	<1 y	<1 y	Pseudophakia OU	NA/NA	-
			III:1 (proband)	F	2 y 4 m	2 y 4 m	2 y 4 m	Anterior subcapsular cataracts OU	19.80/20.05	Concomitant esotropia OU; Posterior synechia of iris OU; Nystagmus OU

*AD, autosomal dominant inheritance; M, male; F, female; y, years; m, months; OU, both eyes; OD, the right eye; OS, the left eye; NA, not available*.

**Figure 3:**
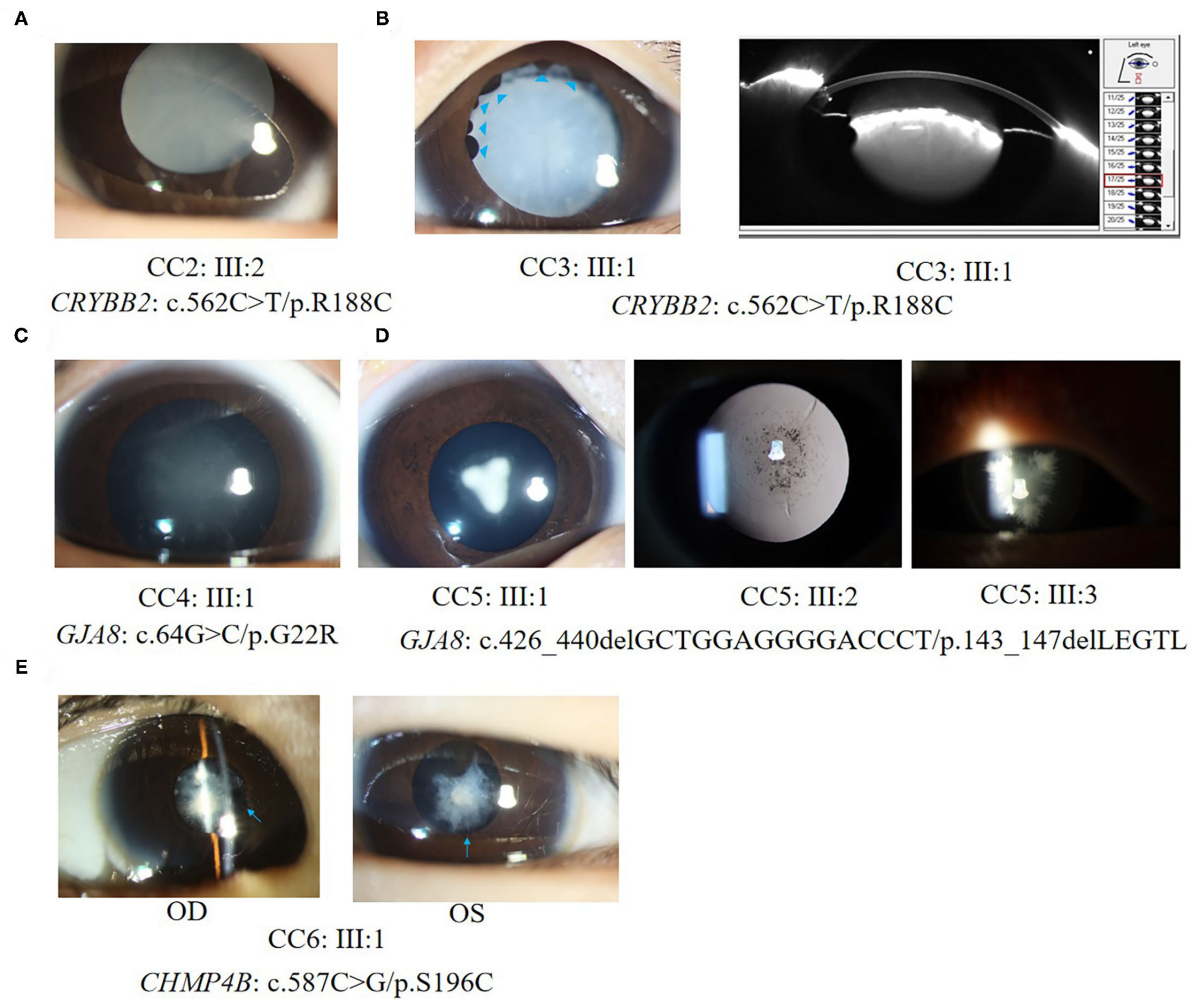
Ocular phenotypes of patients with CCs. **(A)** The total cataract of III:2 in family CC2; **(B)** Left panel: the total cataract and primary iris cysts (blue arrowheads) of III:1 in family CC3; Right panel: the anterior segment image from Pentacam shows the 3 o'clock and 9 o'clock section of cataracts and iris cysts of III:1 in family CC3. **(C)** The fetal nuclear opacities with fuzzy Y-sutural opacity of III:1 in family CC4. **(D)** The Y-sutural and axial embryonal nuclear cataracts with different appearances of III:1, III:2, and III:3 in family CC5. **(E)** The anterior subcapsular cataracts and posterior synechia of iris (blue arrows) of III:1 in family CC6.

All patients in families CC1, CC2, and CC3 carried the same missense mutation, *CRYBB2*: c.562C > T (p.R188C). All probands in the three families had total cataracts in both eyes before 5 months of age. The affected mother of CC1 underwent cataract surgery 23 years ago. The affected mother of CC2 and affected father of CC3 had cataract surgery more than 30 years ago. Therefore, there were no detailed cataract phenotype records. The cataract phenotypes of the probands in CC2 and CC3 are shown in Figures 3A,B. All available patients in CC1 and CC3 had elongated axial lengths in both eyes. At the age of 33, member II:1 in family CC3 had retinal detachment in his left eye due to the long axial length. Nevertheless, the axial lengths of III:2 and III:3 in family CC2 were shorter than the axial lengths of the children in the same age group (18, 19). The corneal diameters and anterior chamber depths of the two patients were normal. Their mother II:2 was also a patient of CCs. She had an elongated axial length of 26.73 mm of the right eye at 31 years old, while her left eye's axial length was normal (23.98 mm). It is worth noting that several iris cysts occurred at the pupillary margin of member III:1 in family CC3 (Figure 3B, left panel). Iris cysts can also be observed in the anterior segment image of the Pentacam rotating Scheimpflug camera at corresponding positions (Figure 3B, right panel). During 3 years of follow-up, this patient's intraocular pressure was in the normal range.

Two different mutations in the *GJA8* gene were separately detected in families CC4 and CC5. The candidate variant of family CC4 is at exon 2 of *GJA8*. It is a base substitution change: c.64G > C (p.G22R). The proband of family CC4 (III:1) was a 4-month-old boy. He had bilateral fetal nuclear opacities with fuzzy Y-sutural opacity (Figure 3C). The mutation detected in family CC5, p.143_147delLEGTL, was a small deletion of five amino acid residues previously reported in another Chinese family (17). Similar to the opacity phenotype of family CC4, members III:1, III:2, and III:3 in family CC5 had Y-sutural and axial embryonal nuclear cataracts with different appearances (Figure 3D). Member III:1 had dense Y-shaped opacities surrounded by punctate embryonal nuclear cataracts. Member III:2 had faint Y-sutural cataracts with bulb and punctate embryonal nuclear cataracts. The white lens opacity at each branch of the Y sutures of member III:3 looked like a feather duster and had dense embryonal nuclear opacities.

In family CC6, a novel missense mutant S196C in the *CHMP4B* gene was detected. The proband had anterior subcapsular cataracts in both eyes with posterior iris synechia (Figure 3E). On the date of the last examination, none of the patients in families CC1–6 had other anterior segment complications after bilateral cataract surgeries.

## Discussion

This study summarized the candidate variants and clinical data of a cohort of Chinese Han patients with CCs and analyzed the genotype-phenotype correlations. Four heterozygous mutations in three genes were found to be associated with CCs in these Chinese families, including three missense mutations and one small deletion. The three missense mutations were in the *CRYBB2, GJA8*, and *CHMP4B* genes. The small deletion was in the *GJA8* gene.

*CRYBB2* encodes the βB2-crystalline protein. It is expressed in the human lens, brain, and retina (20). It is constituted by six exons. The last two amino acid residues of exon 2 and all remaining exons encode four “Greek key” motifs of the βB2-crystalline protein. The mutation R188C is in exon 6 of *CRYBB2*, which results in arginine replacement by cysteine in the fourth Greek key motif of βB2 crystallin. This variant was detected as a *de novo* mutation in three unrelated Chinese families. This might be a mutant hotspot on the gene variation spectrum of *CRYBB2* in the Chinese Han population. This mutation was reported before in a small Chinese family (16). The proband and his affected father in this family had opacities in the posterior pole of the lens (16). In this study, all available patients carrying the R188C mutation had total cataracts within 5 months of age. Another variant at the same amino acid site reported previously was *CRYBB2*: c.563G > A (p.R188H). According to previous reports, patients carrying the R188H mutant had anterior axial embryonal nuclear cataracts in a German family, and anterior axial embryonal nuclear cataracts (a 4-year-old patient) and coralliform cataracts (a 29-year-old patient) in a Chinese family (21, 22).

βB2-crystalline aggregates of different sizes can self-associate to form homodimers or to form heterodimers with other beta-crystallins (23). The R188C change causes a local difference in the surface shape of βB2-crystallin and decreases the charge in that region. It also reduces the polarity and increases the local hydrophobicity of the βB2-crystallin protein. Moreover, the presence of a newly exposed cysteine residue would allow for the formation of intermolecular disulfide bonds under oxidizing conditions (24). This can lead to protein aggregation and cause cataracts (25). In addition, a mutation of the arginine residue identical to histidine in the *CRYBB2* gene has been reported before (21, 22). This underscores the importance of arginine 188. Patients with the R188C mutation had more severe lens opacities and quicker cataract progression than those carrying the variant R188H. Comparing the predicted hydrophobicity of the two missense mutations with the *CRYBB2* wild type showed that both mutations have higher hydrophobicity than the wild type, which might promote the aggregation/fibrillization of the mutated βB2-crystalline. The hydrophobicity of the R188C mutation is higher than that of the R188H mutation. This might substantially lead to quicker progress of opacity development and more severe phenotypes of cataracts. Mutants in *CRYBB2* were also associated with anterior segment dysplasia, including microcornea and glaucoma (26). In this study, a new phenotype of primary iris cysts was observed in the proband of family CC3 (III:1). However, only one patient was found with this phenotype. It is still unclear whether iris cysts are associated with mutations and the pathological mechanism of *CRYBB2*.

The *GJA8* gene encodes Cx50. It is expressed in differentiating lens fibers and persists in mature fibers (27). Connexins form intercellular gap junction channels and hemichannels, which are involved in the passage of intercellular transport of ions and low molecular weight biomolecules (17). The typical structure of connexins includes a short N-terminal cytoplasmic domain, four transmembrane domains (TM1–TM4), two extracellular loops, a cytoplasmic loop domain between TM2 and TM3, and a C-terminal intracellular tail (28). The novel mutation *GJA8*: p. G22R is located in exon 2 at the first amino acid residue of TM1. This substitution replaces a buried uncharged glycine residue with a charged arginine residue. The buried charged residue may reduce the stability of the protein (29). A homologous G22R mutation was detected in the lens opacity 10 (Lop 10) mouse model as a naturally occurring autosomal semidominant mutation (30, 31). The Lop 10 mouse cataract phenotypes are variable, ranging from pulverulent opacities to dense fetal nuclear opacities. In the mouse model, the glycine to arginine substitution fails to form normal gap junctions. It alters the function of connexin 46, which leads to a dominant-negative effect (30, 31). Our study was the first time this mutation was detected in *Homo sapiens* with CCs. The shape of the lens opacity was pulverulent fetal nuclear cataracts with fuzzy Y-sutural opacity, as previously reported in some Lop mouse phenotypes. Another mutation, *GJA8*: p.143_147delLEGTL, is in the CL domain between TM2 and TM3. The same mutation was reported in a Chinese family with bilateral congenital nuclear cataracts in 2016 (17). The five amino acids are arranged with hydrophilic amino acid residues and hydrophobic amino acid residues at intervals (Figure 2B, c). The deletion of this regular arrangement might destabilize the hydrophobic surface and hydrophilic channel of Cx50. Molecular dynamics simulation of this mutation also revealed that the mutant protein disrupts the structural stability of the Cx50 channel, which had a dominant-negative effect similar to the G22R mutant (17).

Mutations in crystallin and connexin genes account for most inherited cataracts (32, 33). *CHMP4B* belongs to the chromatin-modifying protein (CHMP) family. It is one of the components of the endosomal sorting complex required for transport III (ESCRT-III), a complex involved in the degradation of surface receptor proteins and the formation of endocytic multivesicular bodies (34). It is not a common candidate gene of CCs. Previously, only three missense mutations in *CHMP4B* (p. H57R, p. D129V, p. E161K) were found to be associated with posterior subcapsular or posterior polar cataracts (35, 36). In the present study, the patient carrying the S196C mutation had bilateral dense anterior subcapsular cataracts with iris inferior posterior synechia. All four mutations were in the SNF7 domain of the *CHMP4B* gene. It is involved in endosome- and lysosome-associated protein sorting and trafficking (37). Transfection studies of cultured cells revealed that a truncated form of recombinant D129V-*CHMP4B* had an increased capacity to inhibit the release of virus-like particles from the cell surface compared with the wild type (35). It has been suggested that deleterious gain-of-function effects in the ESCRT-III subunit may affect lens transparency (35). The substitution of 196 serine residues with cysteine residues leads to an increase in the local hydrophobicity of chromatin-modifying protein 4b. The exposed cysteine residue may form intermolecular disulfide bonds with other interacting molecules and lead to a dominant-negative effect. Nevertheless, due to the lack of a high identity template, the model confidence of S196C is low. Additional studies are necessary to reveal the detailed pathological mechanism between S196C missense and congenital subcapsular cataracts.

Our research detected four mutations located in the *CRYBB2, GJA8*, and *CHMP4B* genes in six Chinese Han families, including two novel mutations. This expands the mutation and genotype spectrum of the three genes. It can also help with early prenatal diagnosis for families carrying these variants. CCs are widely accepted as a kind of disease with high phenotypic heterogeneity. Nevertheless, in our previous study, it was observed that patients with CCs with lens opacities in the same group (total, anterior, interior, and posterior cataracts) had common anterior segment characteristics (6). In this study, we observed that patients with the same mutations or mutations with similar pathological mechanisms in the same gene were detected within the same group of cataract phenotypes at a similar age. When cataract phenotypes are observed, it would be better to record the patient's age at which they had these lens opacities. This might help reveal the relationship between the phenotypes of CCs and the pathogenic mechanism of candidate genes more clearly. Other ocular phenotypes, including long eye axial lengths and primary cysts of the iris, were observed only in some of the patients. The correlation between these extra lens phenotypes and the function of the mutations needs to be further confirmed.

There were some limitations to this study. First, the number of patients recruited was limited. It might lead to a bias of the genotype-phenotype correlation analysis results. Further exploration should be carried out in a larger cohort or in other populations. Second, the effects of these mutations on protein function were only predicted in silica or deduced from previous functional studies. It is necessary to further study the relationship between the mutant *CHMP4B*:p. S196C and CCs in cell and animal models. This might offer more information for exploring specific molecular mechanisms and potential targets for medical interventions of cataracts.

In summary, by combining WES and bioinformatics analysis technology, we identified four mutations, including two novel mutations, in the *CRYBB2, GJA8*, and *CHMP4B* genes in six Chinese Han families with CCs. This study expanded the mutation and genotype spectrum of the three genes. The variant *CRYBB2*: p. R188C might be a hotspot mutant in the variation spectrum of *CRYBB2* in the Chinese Han population. In the six families, patients carrying the same mutation or mutations in the same gene with similar pathological mechanisms had the same cataract pattern. This result indicates that even though CCs are highly phenotypically heterogeneous diseases, close phenotype-genotype relationships could still be observed between specific cataract phenotypes and mutation sites. Recording patients' ages with the phenotypes of cataracts might better reveal the relationship between CCs and candidate genes. The findings in this study may provide clues for studies in the future to verify the exact roles and specific molecular mechanisms of the three genes in the formation of CC phenotypes. Because the number of patients recruited in this study was limited, larger cohorts and functional studies are needed to further explore the pathogenic mechanisms of these mutations.

## Data Availability Statement

The original contributions presented in the study are included in the article/Supplementary Material, further inquiries can be directed to the corresponding authors.

## Ethics Statement

The studies involving human participants were reviewed and approved by the Institutional Review Board of Zhongshan Ophthalmic Center. Written informed consent was obtained from the individual(s), and minor(s)' legal guardian/next of kin, for the publication of any potentially identifiable images or data included in this article.

## Author Contributions

XW, DW, WC, and HL designed this study. XW, DW, and QW analyzed and interpreted the data for the work. QW, WHua, MD, XZ, DL, ZL, JL, WHu, XLi, XLin, and QZ collected and measured data. XW and DW drafted the work. QW and DL revised it critically for important intellectual content. All authors discussed the results and commented on the manuscript.

## Funding

This research was funded by the Science and Technology Planning Projects of Guangdong Province, Grant No. 2019B030316012 and the National Natural Science Foundation of China, Grant Nos. 81822010, 81770967, and 82000946. The APC was funded by the Science and Technology Planning Projects of Guangdong Province, Grant No. 2019B030316012.

## Conflict of Interest

The authors declare that the research was conducted in the absence of any commercial or financial relationships that could be construed as a potential conflict of interest.

## Publisher's Note

All claims expressed in this article are solely those of the authors and do not necessarily represent those of their affiliated organizations, or those of the publisher, the editors and the reviewers. Any product that may be evaluated in this article, or claim that may be made by its manufacturer, is not guaranteed or endorsed by the publisher.

## Supplementary Material

Supplementary Figure 1 Predicting results of the ProtScale program. **(A)** The predicting result of the hydrophobicity of wild-type βB2-crystallin; **(B)** The predicting result of the hydrophobicity of R188C mutant βB2-crystallin; **(C)** The predicting result of the hydrophobicity of R188H mutant βB2-crystallin.

Supplementary Table 1 Primers that were used in Sanger sequencing.

Supplementary Table 2 Other variants that were detected in the six unrelated congenital cataracts probands.
