# Supplementary figures and images for: Broadening the Mutation Spectrum in GJA8 and CHMP4B: Novel Missense Variants and the Associated Phenotypes in Six Chinese Han Congenital Cataracts Families
Source: Front Med (Lausanne). 2021 Oct 15;8:713284. doi: 10.3389/fmed.2021.713284 (PMC8554029; doi:10.3389/fmed.2021.713284)

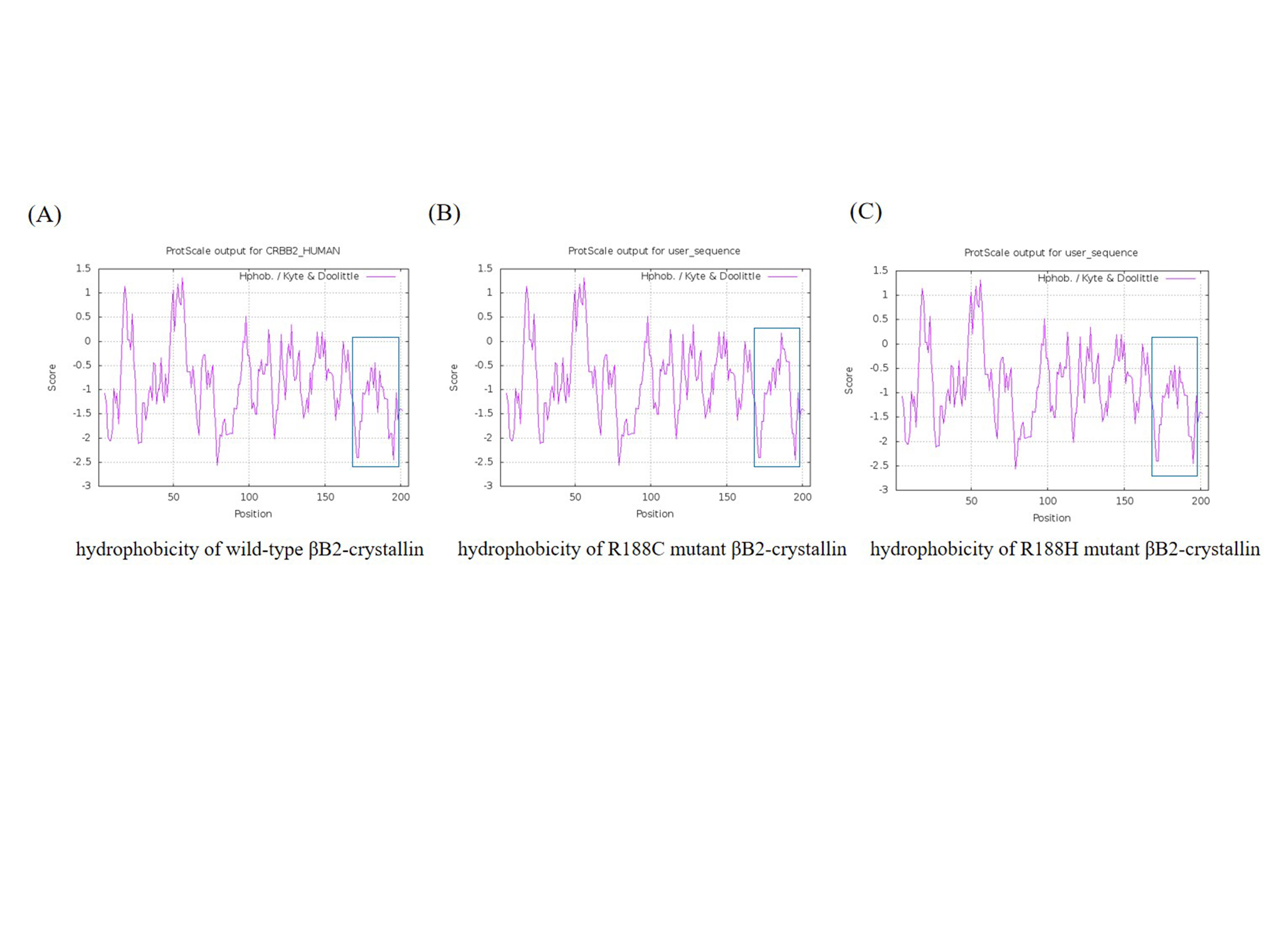

Supplement: Supplementary Figure 1 — Predicting results of the ProtScale program. (A) The predicting result of the hydrophobicity of wild-type βB2-crystallin; (B) The predicting result of the hydrophobicity of R188C mutant βB2-crystallin; (C) The predicting result of the hydrophobicity of R188H mutant βB2-crystallin. [file Image_1.jpg]
