# Supplementary material for: Broadening the Mutation Spectrum in GJA8 and CHMP4B: Novel Missense Variants and the Associated Phenotypes in Six Chinese Han Congenital Cataracts Families
Source: Front Med (Lausanne). 2021 Oct 15;8:713284. doi: 10.3389/fmed.2021.713284 (PMC8554029; doi:10.3389/fmed.2021.713284)
Supplement: Supplementary Table 1 — Primers that were used in Sanger sequencing. [file Table_1.DOCX]

Table S1. Primers that were used in Sanger sequencing.

| Primer name | mRNA | Primer 5'- 3' | Amplicon (bp) | Detected variants |
| --- | --- | --- | --- | --- |
| *CRYBB2*-C562T-F | NM_000496.3 | GGAAGGGTGGGGTGGAAAG | 550 | *CRYBB2:* c.562C>T |
| *CRYBB2*-C562T-*R* | NM_000496.3 | TTCTCATTTCTCTCTCGCTGTCAC |  |  |
| *GJA8*-G64C-F | NM_005267.5 | CCGCTCAGCTCTTGCCTT | 212 | *GJA8*: c.64G>C |
| *GJA8*-G64C-R | NM_005267.5 | TGTTGCACACGAAGTCGGAT |  |  |
| *GJA8*-426_440del-F | NM_005267.5 | ACCGTGCTTTTCATCTTCCG | 846 | *GJA8*: c.426_440delGCTGGAGGGGACCCT |
| *GJA8*-426_440del-R | NM_005267.5 | GGGTCCTGTGCTGATCTTCT |  |  |
| *CHMP4B*-C587G-F | NM_176812.5 | GTAGGAGGCATGACCGCG | 328 | *CHMP4B*: c.587C>G |
| *CHMP4B*-C587G-R | NM_176812.5 | GGGCAAGCTCAGGACACA |  |  |
