# Supplementary material for: Broadening the Mutation Spectrum in GJA8 and CHMP4B: Novel Missense Variants and the Associated Phenotypes in Six Chinese Han Congenital Cataracts Families
Source: Front Med (Lausanne). 2021 Oct 15;8:713284. doi: 10.3389/fmed.2021.713284 (PMC8554029; doi:10.3389/fmed.2021.713284)
Supplement: Supplementary Table 2 — Other variants that were detected in the six unrelated congenital cataracts probands. [file Table_2.DOCX]

Table S2. Other variants that were detected in the 6 unrelated congenital cataracts probands.

| Pro-  bands  ID | Gene | mRNA | Nucleotide Change | Amino Acid Change | State | Computational Prediction | | | | Allele Frequency in | | | Cosegre-gation | Excluded Criteria | Reported |
| --- | --- | --- | --- | --- | --- | --- | --- | --- | --- | --- | --- | --- | --- | --- | --- |
|  |  |  |  |  |  | PPH-2/BDGP | SIFT | PROVEAN | MutationTaster | 1000G | dbSNP | gnomAD |  |  |  |
| CC1 | HSD17B4 | NM_  001199292 | c.533delC | p.A178fs | Het | - | - | - | - | - | - | - | N | a,c | Novel |
| CC1 | EPG5 | NM_020964 | c.3240-2A>G | - | Het | SSA | - | - | D | - | - | - | N | a,c | Novel |
| CC1 | CRYBB1 | NM_001887 | c.326T>A | p.F109Y | Het | B | T | N | D | - | - | - | N | a,c | Novel |
| CC1 | SREBF2 | NM_004599 | c.2831A>G | p.H944R | Het | PrD | T | D | D | - | - | - | Y | c | Novel |
| CC1 | BCOR | NM_  001123383 | c.1888G>A | p.E630K | Het | PrD | D | N | D | - | - | - | N | a,c | Novel |
| CC1 | USP9X | NM_  001039590 | c.2779G>A | p.V927I | Het | B | T | N | N | - | - | 0.000006 | N | a,c | Novel |
| CC2 | CC2D2A | NM_  001080522 | c.1810G>A | p.G604S | Het | B | T | N | N | - | 0.000013 | 0.000029 | Y | b,c | rs747935651 |
| CC2 | TFAP2A | NM_  001032280 | c.299G>A | p.G100E | Het | B | T | N | D | - | - | 0.000008 | N | a,c | Novel |
| CC2 | RECQL4 | NM_004260 | c.440C>T | p.P147L | Het | B | - | - | - | - | 0.000011 | 0.000014 | N | a,c | rs759034699 |
| CC2 | PMPCA | NM_  001282944 | c.67G>T | p.D23Y | Het | B | T | D | D | - | 0.000071 | 0.000004 | N | a,c | rs777534357 |
| CC3 | HOXD13 | NM_000523 | c.46G>A | p.G16S | Het | B | T | N | D | - | - | - | Y | b,c | Novel |
| CC3 | WFS1 | NM_  001145853 | c.2067_2076  del10 | p.L689fs | Het | - | - | - | - | - | - | - | N | a,c | Novel |
| CC3 | SLC4A4 | NM_003759 | c.2176G>A | p.V726I | Het | B | T | N | D | - |  | 0.000020 | Y | b,c | rs150967020 |
| CC3 | GBA2 | NM_  001330660 | c.1604A>G | p.N535S | Het | PrD | D | D | D | - | - | 0.000004 | N | a,c | Novel |
| CC4 | SLC2A1 | NM_006516 | c.310T>G | p.F104V | Het | B | T | N | D | - | - | - | Y | b | Novel |
| CC4 | COL1A2 | NM_000089 | c.814A>G | p.N272D | Het | B | T | N | N | - | - | - | Y | b | Novel |
| CC5 | HOXD13 | NM_000523 | c.8G>T | p.R3L | Het | B | D | N | N | - | - | - | N | a,c | Novel |
| CC5 | ADAM9 | NM_003816 | c.639T>G | p.Y213X | Het | - | - | - | - | - | - | - | N | a,c | Novel |
| CC5 | ADAM9 | NM_003816 | c.641T>G | p.V214G | Het | PrD | D | D | D | - | - | - | N | a,c | Novel |
| CC5 | ZNF408 | NM_024741 | c.2T>C | p.M1T | Het | B | D | N | D | - | - | 0.000008 | N | a,c | Novel |
| CC5 | COL18A1 | NM_030582 | c.13C>T | p.P5S | Het | P | D | D | D | - | - | 0.000004 | N | a,c | Novel |
| CC5 | CRYBA4 | NM_001886 | c.76C>T | p.R26W | Het | B | D | D | D | - | 0.000029 | 0.000012 | N | a,c | rs150427830 |
| CC6 | MYO7A | NM_000260 | c.359G>T | p.R120L | Het | B | D | D | D | - | - | - | N | a,c | Novel |
| CC6 | MAN2B1 | NM_000528 | c.1988G>C | p.R663T | Het | PrD | D | D | D | - | - | - | N | a,c | Novel |
| CC6 | RPGR | NM_000328 | c.1777G>A | p.A593T | Het | B | T | N | N | - | - | 0.000006 | N | a,c | Novel |

Note: PPH-2/BDGP= Polyphen-2/Splice Site Prediction program with Neural Network; 1000G=1000 Genomes database; Het= heterozygous; SSA= splicing site abolished; B= benign; PrD= probably damaging; D= damaging in SIFT and deleterious in PROVEAN; T= tolerated; N= Neutral; -= not applicable.

The exclude critera of potential pathogenic mutations:

a, The mutations were not segregated with the disease in their families.

b, The mutations were predicted to be benign in three of the four computational prediction tools.

c, Other more likely pathogenic mutations were detected and are listed in Table 1.
